# Functional Consequences of Cell Type-Restricted Expression of Laminin α5 in Mouse Placental Labyrinth and Kidney Glomerular Capillaries

**DOI:** 10.1371/journal.pone.0041348

**Published:** 2012-07-20

**Authors:** Sung Tae Kim, Tracy L. Adair-Kirk, Robert M. Senior, Jeffrey H. Miner

**Affiliations:** 1 Renal Division, Washington University School of Medicine, St. Louis, Missouri, United States of America; 2 Division of Pulmonary and Critical Care Medicine, Washington University School of Medicine, St. Louis, Missouri, United States of America; 3 Department of Cell Biology and Physiology, Washington University School of Medicine, St. Louis, Missouri, United States of America; The University of Hong Kong, Hong Kong

## Abstract

The labyrinth is the highly vascularized part of the rodent placenta that allows efficient transfer of gases, nutrients, wastes, and other molecules between the maternal and embryonic circulations. These two blood compartments are separated by blastocyst-derived trophoblasts and endothelial cells with an intervening basement membrane that contains laminin and other typical basement membrane components. Previously we reported that the labyrinth of laminin α5 knockout (*LMα5*−/−) embryos exhibits reduced vascularization and detachment of endothelial cells from the basement membrane, which normally contains LMα5. As very little is known about the origin of this vascular basement membrane, we investigated the cellular requirements for LMα5 expression in the mouse placental labyrinth. By fluorescence-activated cell sorting and RT-PCR we confirmed that both endothelial cells and trophoblasts normally express LMα5. Using Cre-loxP technology and doxycycline-mediated gene expression, we generated genetically mosaic placentas in which either the trophoblasts or the endothelial cells, but not both, expressed LMα5. We found that the overall architecture of the labyrinth was normal as long as one of these two cell types expressed LMα5, even if it was transgene-derived human laminin α5. These results suggest that laminin trimers containing α5 that are synthesized and secreted by endothelium or by trophoblasts are capable of integrating into the basement membrane and promoting normal vascularization of the placenta. Additional studies showed that endothelium-expressed human LMα5 can support vascularization of the kidney glomerulus, consistent with previous studies using a tissue grafting approach.

## Introduction

Basement membranes (BMs) are thin sheets of specially organized extracellular matrix proteins associated with many different cell types, including all endothelial and epithelial cells. BMs promote proliferation and survival, provide signals, pathways, and barriers for cell migration, and are responsible for establishing and maintaining compartmentalization within tissues. All BMs contain four major classes of proteins: laminin (αβγ heterotrimers), collagen IV (α chain heterotrimers), nidogen, and heparan sulfate proteoglycan (both monomers).

Because there are numerous protein isoforms within these classes distributed in BMs in distinct cell- and tissue-specific fashions, all BMs are not alike. In many cases, differences in BM composition have been shown to contribute to their functional specificities. For example, the synaptic cleft BM at neuromuscular junctions contains the laminin α2β2γ1 (LM-221), laminin α4β2γ1 (LM-421) and laminin α5β2γ1 (LM-521) heterotrimers, whereas the extrasynaptic BM contains primarily laminin α2β1γ1 (LM-211) [1]. In knockout mice lacking LMβ2, the synaptic laminin trimers cannot assemble, resulting in severe defects in differentiation, structure, and function of the neuromuscular synapse, despite substitution by other laminin trimers [1], [2].

**Figure 1:**
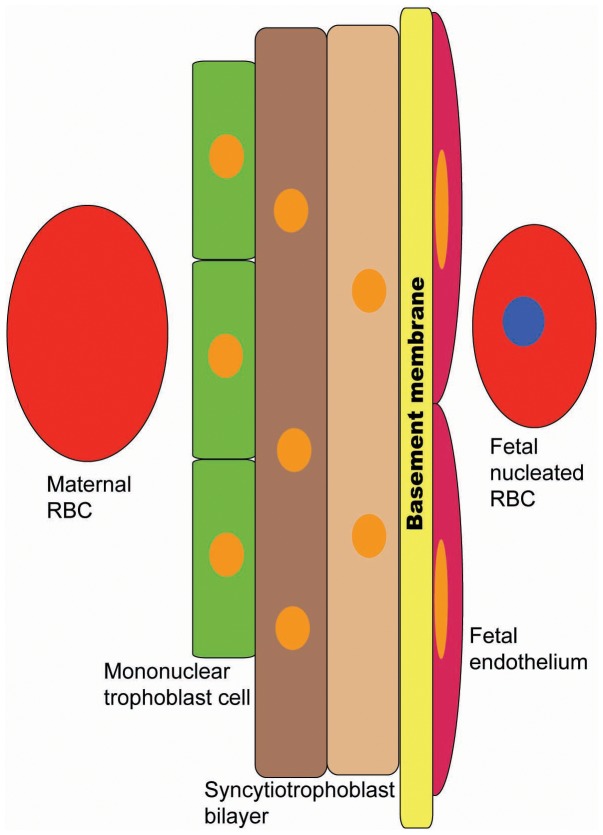
Schematic diagram of the barrier between the maternal and embryonic vasculatures within the placental labyrinth. The placental endothelial basement membrane, which normally contains LMα5, lies between the fetal endothelium and the trilaminar trophoblast cellular structure. Mononuclear trophoblasts line the maternal blood spaces, whereas the other two trophoblast layers are syncytial due to cell-cell fusion. Maternal red blood cells (RBCs) lack nuclei, whereas fetal RBCs retain nuclei until late gestation.

Each of the five mammalian laminin α chains contains a large COOH-terminal laminin globular (LG) domain that harbors binding sites for cell surface receptors, such as integrins and dystroglycan [3]. Different affinities for different receptors likely contributes a significant degree of specificity to laminin trimers and thus to BM function. The LMα5 chain is widely expressed in mouse tissues [4]. Mutation of *Lama5 (LMα5)* in mice causes a diverse array of complex developmental defects and perinatal lethality [5]–[13]. One of the best-characterized defects is in the formation of kidney glomeruli, highly vascularized structures required for filtration of the blood. In the absence of LMα5, which is expressed by both glomerular visceral epithelial cells (podocytes) and endothelial cells [14], the glomerular basement membrane (GBM) between them breaks down, and vascularization fails [6]. Another striking defect observed in the absence of LMα5 is in the labyrinth of the placenta. The placental labyrinth is the highly vascularized part of the placenta where the bidirectional transfer of gases, nutrients, wastes, and other molecules between the maternal and embryonic circulations occurs [15]. In the hemochorial mouse placenta, the barrier between the maternal blood and the embryonic vasculature is formed by three layers of embryo-derived trophoblasts, an endothelial BM, and an embryo-derived endothelium (Fig. 1) [16]. The labyrinth is grossly undervascularized in *LMα5* null mutants, and the vessels that do form are larger caliber compared to control. In addition, fetal placental endothelial cells lose adhesion to the BM, which normally contains LMα5. Together with the fact that *LMα5*−/− embryos are smaller than controls after E14.5, we previously suggested that the abnormalities in the labyrinth lead to placental insufficiency [5]. LMα5 is also a component of BMs in human placenta [17], where it likely plays a similarly important role in placentation, although no defects in human LMα5 (hLMα5) function have yet been reported.

Here we investigated the cellular requirement for LMα5 expression in the mouse placental labyrinth using conditional and constitutive *LMα5* mutant alleles, as well as Cre, Cre-activated reverse tetracycline transactivator (rtTA), and hLMα5 transgenes. Our results suggest that both trophoblasts and endothelial cells normally contribute LMα5-containing trimers to the endothelial BM, and that expression by either cell is sufficient for normal placentation. In addition, we confirmed previous tissue grafting studies [18] showing that endothelial LMα5 expression is sufficient for vascularization of kidney glomeruli.

## Results

### Expression of Laminin Chains in the Placenta

**Figure 2:**
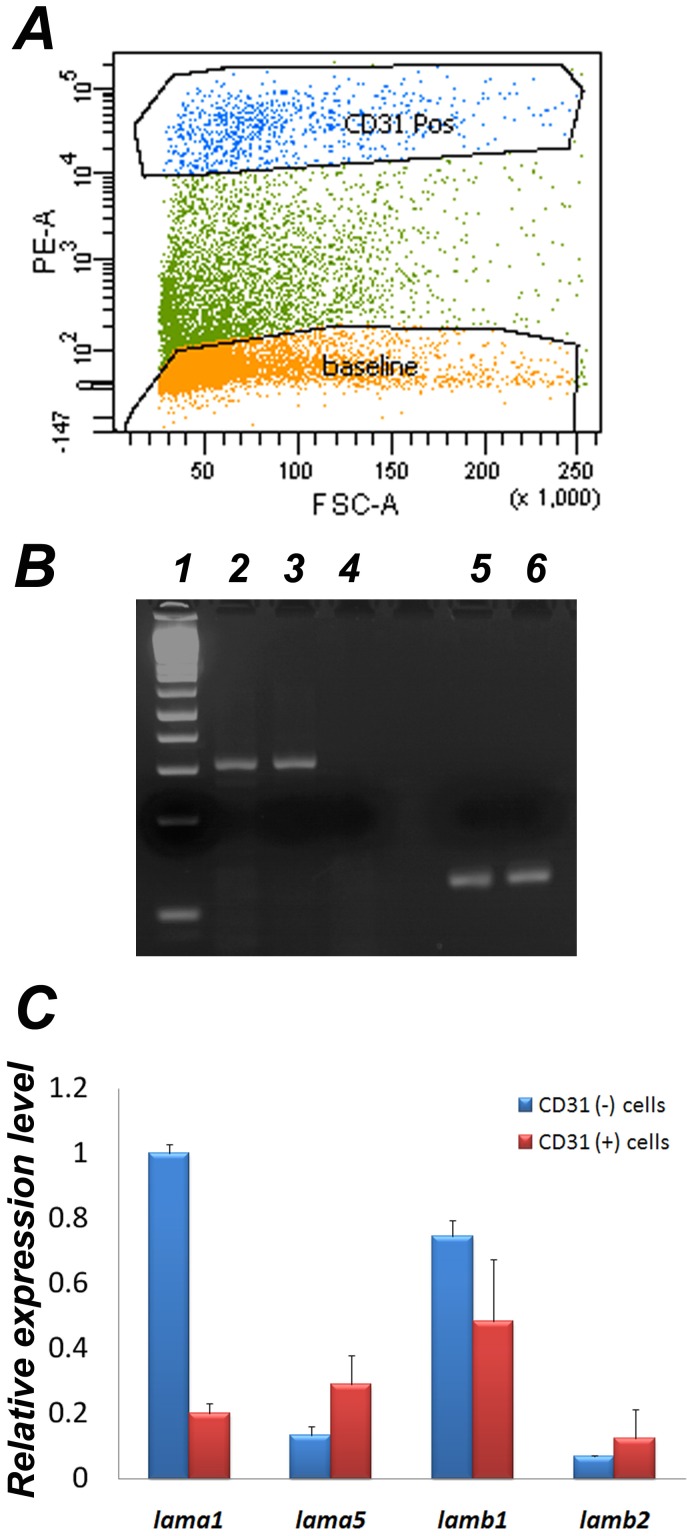
LMα5 is expressed in both endothelial cells and trophoblasts in the normal placenta. (A) Fluorescence-activated cell sorting was performed on dissociated E18.5 wild-type labyrinth cells after staining with a phycoerythrin (PE)-conjugated CD31/PECAM antibody. CD31+ (endothelial cell) and CD31− (trophoblast; indicated as baseline) populations were collected. (B) RT-PCR using RNA prepared from the two cell types showed that LMα5 was expressed in both: Lane 1, DNA marker; 2 and 3, LMα5 in CD31(−) and (+) cells, respectively; 4, negative control; 5 and 6, GAPDH in CD31(−) and (+) cells, respectively. (C) RNA was subjected to real time RT-PCR to quantitate the levels of laminin α1 (lama1), α5 (lama5), β1 (lamb1), and β2 (lamb2) mRNAs. Error bars represent standard deviations.

Although some classes of endothelial cells have been shown to express LMα5, not all do so [19]. To directly investigate whether labyrinth-derived endothelial cells and/or trophoblasts normally express LMα5 and other laminin chains found in the placenta [20], we used fluorescence activated cell sorting (FACS) to isolate endothelial (CD31-positive) and non-endothelial (CD31-negative) cell populations from the normal placental labyrinth (schematized in Fig. 1) after its dissociation into single cells (Fig. 2A). RNAs were prepared from these isolated cells and subjected to quantitative real-time RT-PCR for laminin α5, α1, β1, β2, and GAPDH expression (Fig. 2B,C). The results showed that both populations of cells express each of these laminin chains, but that trophoblasts (CD31-negative cells) express more laminin α1 and β1 than α5 and β2, whereas endothelial (CD31-positive) cells express more laminin α5 and β1 than α1 and β2. The fact that *LMβ2*−/− embryos have normal placental labyrinths and are indistinguishable from control littermates at birth (JHM, unpublished studies and [21]) suggests that the β1-containing trimers are sufficient for promoting normal placentation. Although these studies did not address the origin of laminin α2 and α4 in the labyrinth [20], no placental defects have been reported in the respective knockout mice [22]–[24].

### Consequences of Cell Type-Restricted Expression of LMα5 in the Labyrinth

**Figure 3:**
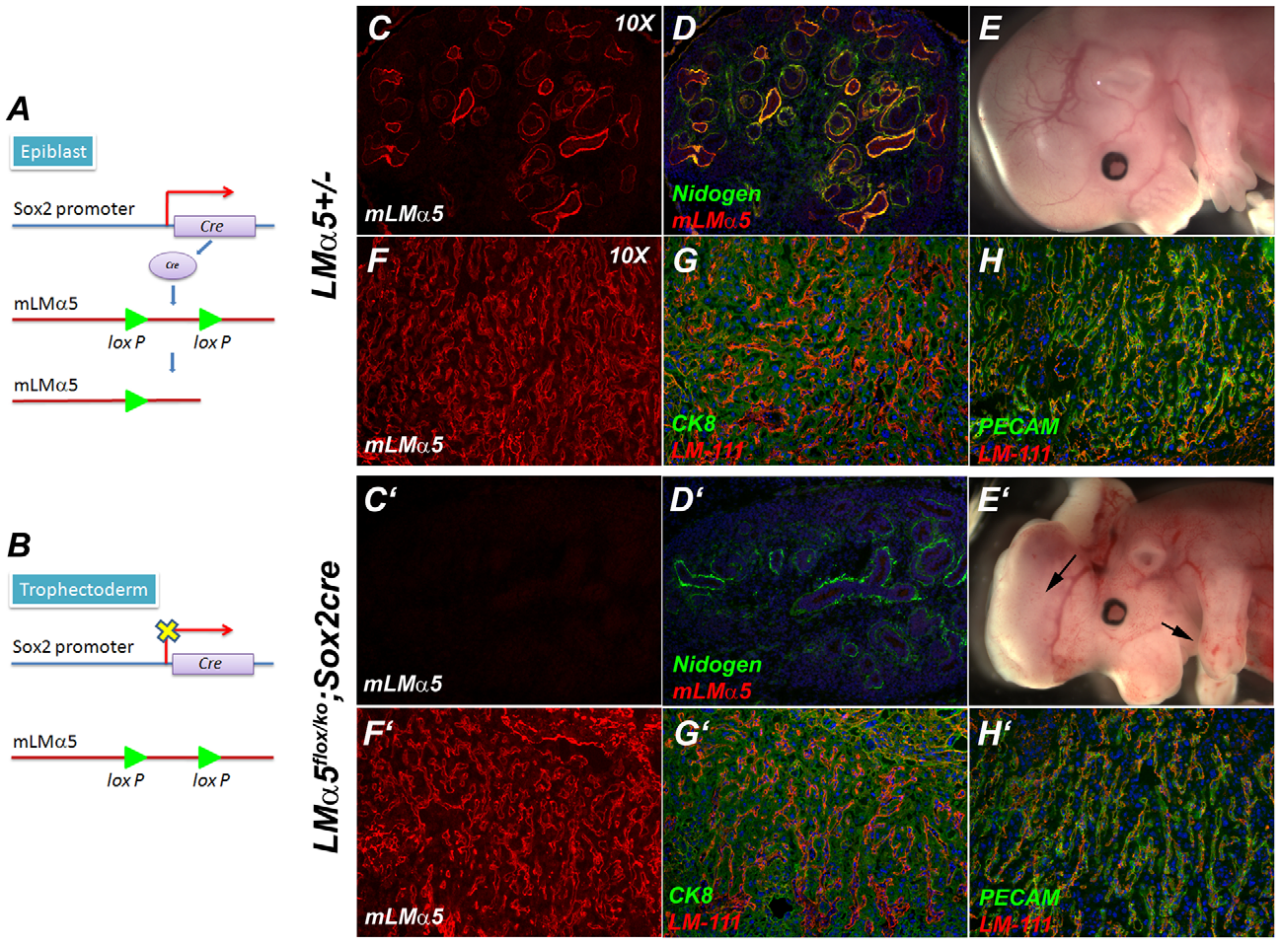
Mosaic placental labyrinths containing wild-type trophoblasts and *LMα5*−/− endothelial cells show LMα5 deposition and normal vascularization. (A, B) Schematic diagrams of the strategy for conditional mouse *LMα5* mutation. Using the Cre/loxP system, we generated *LMα5^flox/ko^*; Sox2Cre embryos. Sox2cre, when inherited from a male, is active in epiblast, but not in trophectoderm. Thus, epiblast-derived cells (A), which include the embryo proper as well as extraembryonic endothelial cells, are not able to synthesize LMα5, but trophoblasts, which derive from trophectoderm (B), can. (C–H; C′–H′) Analysis of LMα5 expression and tissue architecture in control (top rows) and *LMα5^flox/ko^*; Sox2cre mutant (bottom rows) embryos. LMα5 was not expressed in the kidney of *LMα5^flox/ko^*; Sox2cre embryos (C′; counterstained with anti-nidogen in D′; compare with control, C and D), which show developmental abnormalities typical of *LMα5*−/− embryos (E′; arrows indicate exencephaly and syndactyly) not seen in control (E). In contrast, LMα5 was present in the placental labyrinth of *LMα5^flox/ko^*; Sox2cre embryos (F′) and of control (F), and placental LM-111 and PECAM expression and localization were similar to those observed in control *LMα5+/*− placenta (G–H, G′–H′). Cytokeratin 8 (CK8) was used to identify trophoblasts (G, G′).

To determine the cellular requirements for LMα5 expression in the mouse placental labyrinth, we used Cre/LoxP and doxycycline-inducible systems to generate mosaic placentas in which either the trophoblasts or the endothelial cells, but not both, were capable of expressing LMα5. We used two distinct approaches. First, to generate placentas with normal trophoblasts and *LMα5* null endothelial cells, we took advantage of the selective expression pattern of the Sox2Cre transgene [25]. When this gene is transmitted by the sire, it is expressed in the epiblast (Fig. 3A), which gives rise to the embryo proper and to the allantois, from which originate the extraembryonic endothelial cells of the labyrinth [26]; however, Sox2Cre is not expressed in the trophectoderm (Fig. 3B), which gives rise to the trophoblasts.

We mated *LMα5+/−*;Sox2Cre males with *LMα5^fl^* females to generate *LMα5^fl/−^*;Sox2Cre (mutant) embryos and littermate controls. At E14.5, mutant embryos (at least five from different litters were examined in detail) showed the typical *LMα5* null embryonic phenotype—partially penetrant exencephaly and syndactyly (Fig. 3E′; compare to E) associated with a lack of LMα5 (Fig. 3C′,D′; compare to C,D), although BMs were generally positive when immuno-stained for nidogen (Fig. 3D′). In contrast, we detected abundant LMα5 protein in placental labyrinth BMs, and the overall architecture of the labyrinth was similar to that of control littermates (Fig. 3F–H, F′–H′); there was an extensive network of PECAM-positive small caliber vessels, and most maternal blood spaces, which are lined by cytokeratin 8-positive trophoblasts, were juxtaposed to embryonic vessels with BMs that stained for LM-111. These results suggest that laminin trimers containing α5 that are synthesized and secreted by trophoblasts are capable of integrating into the BM and promoting normal vascularization of the placenta, but they are not sufficient to rescue *LMα5−/−* phenotypes within the embryo.

**Figure 4:**
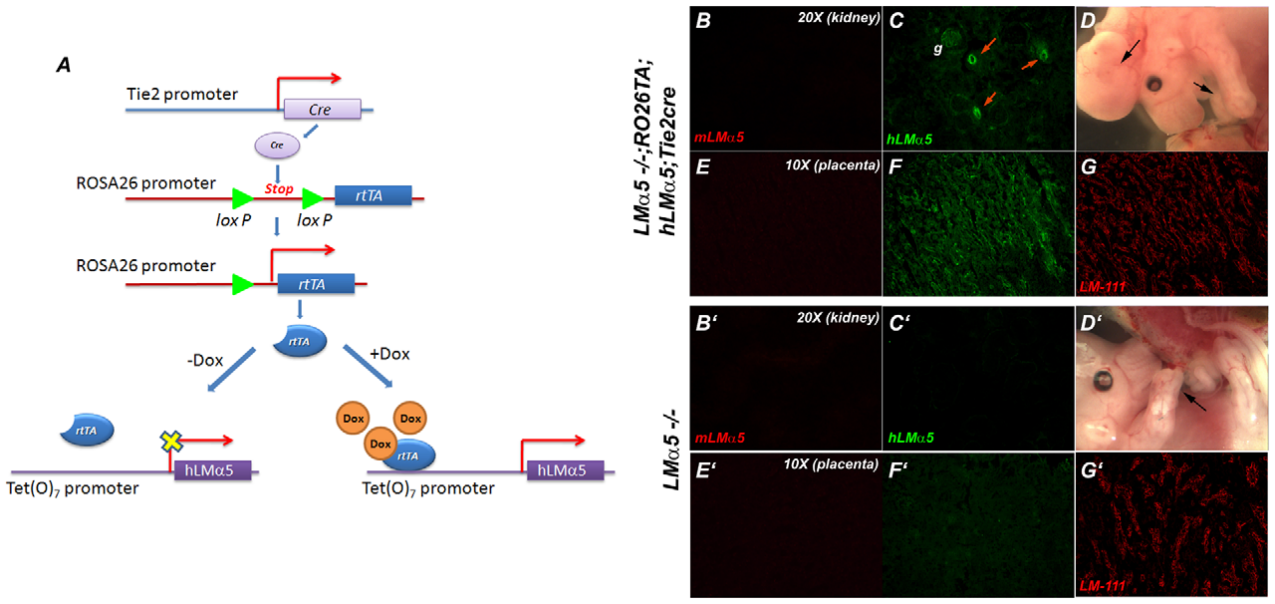
Mosaic placental labyrinths containing *LMα5*−/− trophoblasts and hLMα5-expressing endothelial cells show hLMα5 deposition and normal vascularization. (A) Schematic diagram of the strategy for forcing expression of hLMα5 in endothelial cells on the *LMα5−/−* background. Cre recombinase driven by the Tie2 promoter removes a floxed STOP located between the *Rosa26* promoter and the reverse tetracycline transactivator (rtTA). rtTA binds and activates the tetracycline-inducible TetO_7_ promoter in the presence of doxycycline, thereby driving transcription of the hLMα5 cDNA in endothelial cells. (B–G) *LMα5−/−;ROSA26TA;hLMα5;Tie2cre* embryos (top panels) were compared with *LMα5−/−* embryos (bottom panels). Mouse LMα5 was undetectable in kidney (B, B′) or placenta (E, E′). Human LMα5 was detected in both kidney and placental vasculatures of *LMα5−/−;ROSA26TA;hLMα5;Tie2cre* embryos (C, F) but not of *LMα5−/−* embryos (C′,F′), both of which show the typical *LMα5* null phenotype (D, D′). Expression of hLMα5 in endothelial cells was associated with a normalized placental labyrinth architecture, demonstrated by the LM-111 antibody staining pattern (compare G and G′).

In the second approach, we utilized a combination of transgenes and mutations to perform the converse experiment. We used the endothelial cell-specific Tie2Cre transgene to activate expression of the reverse tetracycline transactivator (rtTA), which had been knocked into the *Rosa26* locus preceded by a floxed STOP (genotype *RO26TA*). In the presence of doxycycline, this rtTA drives expression of the TetO_7_-regulated hLMα5 transgene (Fig. 4A). When these three loci are present in genetically *LMα5*−/− embryos exposed to doxycycline, all endothelial cells should express hLMα5, and there is no other source of α5 (either mouse or human).

**Figure 5:**
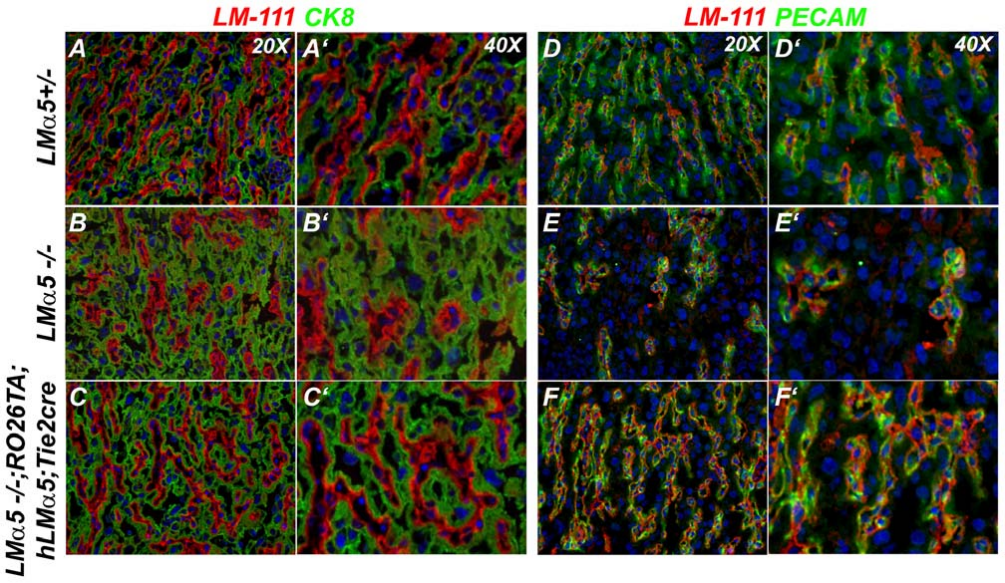
Analysis of placental labyrinth vasculature at E14.5. Frozen sections of placenta were stained with antibodies to LM-111 to label all basement membranes, to cytokeratin 8 (CK8) to label trophoblasts (green in A–C, A′–C′), and to PECAM to label endothelial cells (green in D–F, D′–F′). The reduced vascular complexity in the *LMα5−/−* labyrinth (B, E) was rescued and made similar to normal (A, D) by hLMα5 secretion from *LMα5−/−;ROSA26TA;hLMα5;Tie2cre* endothelial cells (C, F) exposed to doxycycline.

**Figure 6:**
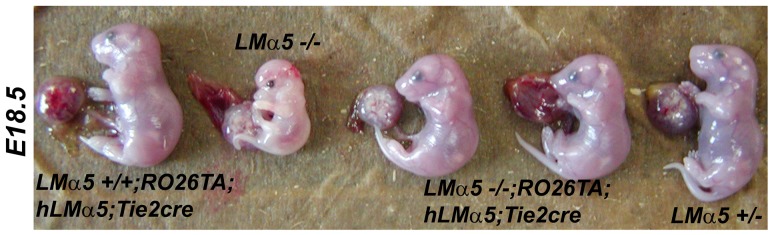
Phenotype of various *LMα5* mutant and control embryos at E18.5. Genotypes are indicated. The mother was fed doxycycline beginning at E0.5. Endothelial expression of hLMα5 in *LMα5−/−;RO26Ta;hLMα5;Tie2cre* embryos resulted in a larger (though still not quite normal) embryo size compared to the nontransgenic *LMα5−/−* embryo.

Seven embryos of the appropriate genotypes from four litters, along with *LMα5*−/− and normal control embryos, were identified by PCR and studied. At E14.5, *LMα5*−/−;*RO26TA*;*hLMα5*;*TIE2Cre* embryos showed the typical *LMα5* null phenotype (Fig. 4D; compare to D′) and lacked mouse LMα5 (Fig. 4B,E; compare to B′,E′). As expected from the approach, hLMα5 was detected in embryonic endothelial BMs (Fig. 4C; compare to C′). Human LMα5 was also present in placental labyrinth BMs (Fig. 4F; compare to F′), and this resulted in apparently normal placentation, as determined from the pattern of LM-111 staining (Fig. 4G and Fig. 5C,C′,F,F′), which was similar to the control (Fig. 5A,A′,D,D′). However, this was in stark contrast to the LM-111 staining pattern in the *LMα5* null placenta that did not express hLMα5 (Fig. 4G′ and Fig. 5B,B′,E,E′). The apparently normal placental labyrinth of transgenic *LMα5−/−* embryos was associated with a larger but not quite normal embryo size at E18.5 (Fig. 6); this could be due to rescue of placental insufficiency, but might also stem from an overall healthier vasculature within the embryo itself. Nevertheless, with the exceptions noted below, the typical developmental defects of *LMα5*−/− embryos were still present, including syndactyly, partially penetrant exencephaly (Figs. 4D, 6), and an absent pleural basement membrane in the lung (not shown). Together, these results suggest that endothelium-derived LMα5 can support normal placentation. And combined with the RT-PCR data (Fig. 2), results from the mosaic labyrinth studies suggest that trophoblasts make LM-111 and LM-511, whereas endothelial cells make primarily LM-511 and LM-521.

### Consequences of Endothelium-Specific Expression of LMα5 in the Kidney Glomerulus

**Figure 7:**
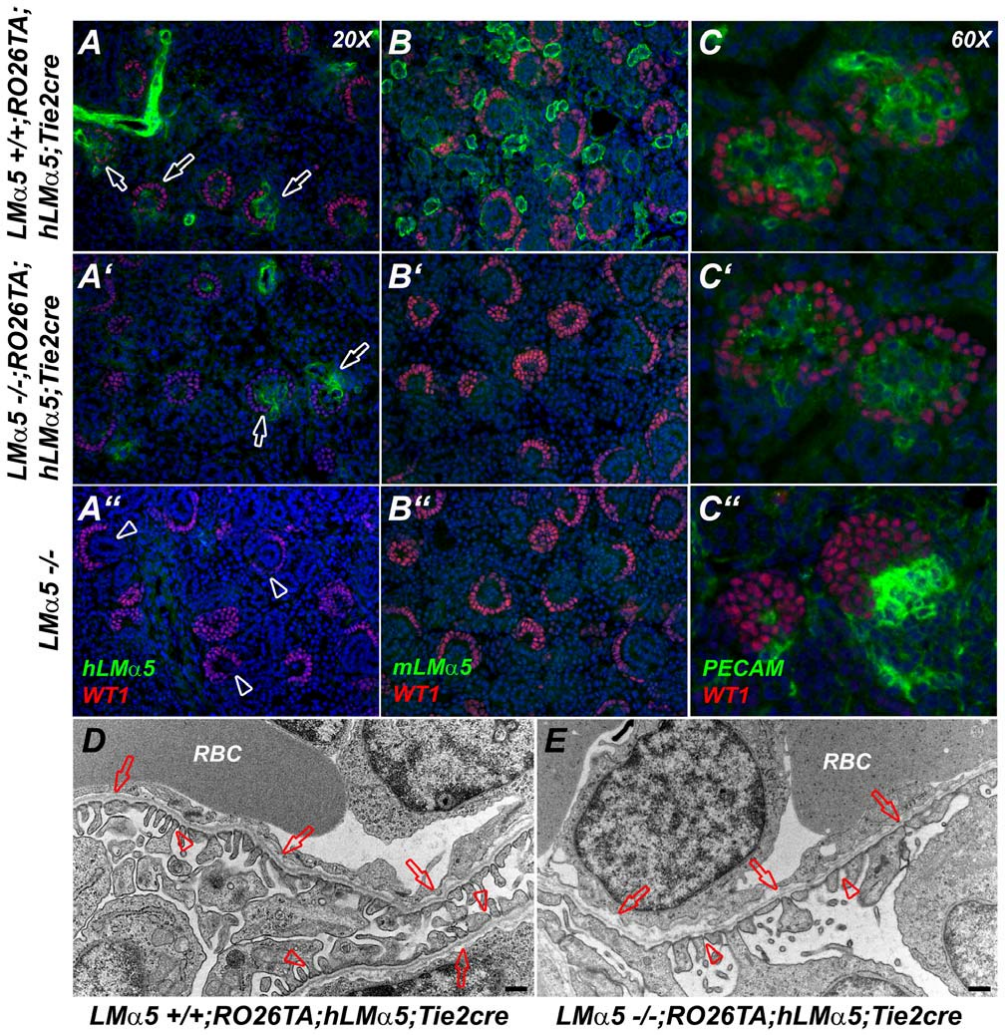
Expression of hLMα5 in glomerular endothelial cells rescues glomerular vascularization in *LMα5−/−* kidney. (A,B) Analysis of human (A) and mouse (B) LMα5 expression (green) relative to WT1 (red), which stains podocyte nuclei at E18.5. Human LMα5 is visible primarily in the glomeruli (open arrows) and in arterioles in embryos carrying the transgenes (A,A′) but is absent from the mutant lacking the transgenes (A″; open arrowheads indicate glomeruli). (C) Status of glomerular vascularization was revealed by PECAM (green) and WT1 (red) double staining. PECAM-positive endothelial cells were properly localized inside glomeruli when either mouse or hLMα5 or both were present in the GBM (C, C′), but glomerulogenesis failed in the absence of LMα5 (C″). (D,E) Transmission electron microscopic analysis of glomeruli in control (D) and rescued mutant (E) kidney reveals that both have an intact GBM (arrows), capillaries containing red blood cells (RBCs), and podocytes with foot processes (arrowheads). Bars in D and E are 500 nm.

We have previously shown that vascularization of the kidney glomerulus is defective in *LMα5* null embryos due to breakdown of the GBM, disorganization of podocytes, and failure of endothelial and mesangial cells to establish glomerular capillaries [6]. However, when embryonic *LMα5*−/− kidneys are grafted into newborn WT kidneys, invading WT endothelial cells can supply LMα5-containing trimers and rescue glomerular vascularization [18]. Here we studied glomerulogenesis at E14.5 (not shown) and E18.5 in several *LMα5* null embryos with endothelial cell-specific expression of hLMα5 (Fig. 7A′–C′) as compared to controls with hLMα5 expression (Fig. 7A–C) and *LMα5−/−* embryos without transgene expression (Fig. 7A″–C″). Similar to the grafted *LMα5* null kidneys infiltrated by wild-type endothelial cells [18], it appears that glomerular endothelial cell expression of hLMα5 (Fig. 7A′) was sufficient to rescue glomerulogenesis, based upon the proper localization of PECAM-positive endothelial cells adjacent to WT1-positive podocytes in the control and “rescued” glomeruli (Fig. 7C,C′); this clearly contrasts with the *LMα5*−/− kidney's avascular “glomeruli” (Fig. 7C″). Rescue of glomerulogenesis was associated with deposition (albeit weak) of hLMα5 in the GBM (Fig. 7A,A′). Finally, transmission electron microscopic analysis of the glomerular capillary wall revealed that endothelial cell-derived hLMα5 promoted maintenance of GBM architecture, successful glomerular vascularization, and even podocyte foot process formation, all of which were comparable to the control (Fig. 7D,E). These results lend support to our previous conclusions regarding the ability of endothelial-cell derived LMα5 to promote glomerulogenesis when podocytes are unable to express it [18], although the glomerular filter eventually becomes leaky to plasma albumin in the absence of podocyte LMα5 expression [27].

## Discussion

The murine placental labyrinth is a highly vascularized organ tailored for the transfer and transport of nutrients, wastes, and gases between mother and embryo [15]. In humans, placental insufficiency and the resulting intrauterine growth restriction are important health problems that can develop due to defects in the structure or function of the placental vasculature [28]. Although the placentas of mice and humans exhibit many differences [29], one similarity is that both the mouse labyrinth and the human chorionic villi, the major sites of materno-fetal exchange, are enriched in BMs, due in part to the density of fetal blood vessels.

We previously showed that mice lacking LMα5 exhibit dramatically impaired vascularization of the placental labyrinth [5]; there are many maternal blood spaces but fewer fetal blood vessels juxtaposed to them than in controls (ref. [20] and Figs. 3 and 5). Thus, there is no apparent defect in the ability of *LMα5−/−* trophoblasts to invade the deciduum and form maternal blood spaces through branching morphogenesis. However, we cannot rule out a contribution of maternal/decidual LMα5 to promoting this invasion.

The placental vasculature is attenuated in the absence of LMα5, despite the fact that the laminin α1, α2, and α4 chains are all present in the BM [20]. In contrast, global mutation of *LMα2* and *LMα4* has not been reported to cause placental defects [22], [23], suggesting that LMα5 has a non-redundant role in placentation. Moreover, structure-function analysis of the LMα5 COOH-terminal LG domain, which has five segments, revealed that the LG1–2 segment is required for promoting normal placental vascularization, and that the analogous LG1–2 segments of LMα1 could not compensate [20]. Interestingly, one of the first monoclonal antibodies known to be made to hLMα5, clone 4C7, was generated by immunizing mice with laminins purified from human placenta [30]. Directed efforts aimed at defining the laminin composition of human placental villous BMs revealed that as in mouse, laminin α1, α2, and α5 are present; LMα4 was not investigated [31]. The possibility therefore exists that, as in mouse, LMα5 plays a critical role in vascularization of the human placenta.

Here we focused on investigating the cellular requirements for LMα5 expression in the mouse labyrinth using mutant *LMα5* alleles and various transgenes to generate mosaic placentas. Moreover, the strategy we used also facilitated studies of mosaic kidney glomeruli. In placenta we found that expression of LMα5 either in trophoblast or in endothelium was sufficient for apparently normal vascularization, despite the fact that α5 is normally expressed in both cell types. Similar studies in kidney glomeruli showed that expression of LMα5 solely in endothelial cells was sufficient to rescue glomerulogenesis in *LMα5*−/− embryos, as predicted from our previous grafting studies [18]. Thus, endothelial LMα5 expression is sufficient for function of two different vascular BMs that are normally co-synthesized by a flanking cell of a different type—trophoblasts in the labyrinth (Figs. 2 and 3) and podocytes in the glomerulus [14], [27]. In contrast, other defects that we have previously studied in detail in *Lama5−/−* embryos, including defects in neural tube closure, digit septation, and lung lobe septation, were not ameliorated by forced endothelial cell-specific expression of LMα5. This was expected, as the presumed mechanisms leading to these defects do not involve endothelial basement membranes [5], [7].

What is the function of LMα5 in the placental labyrinth? Histopathology suggests there is a defect both in branching morphogenesis/angiogenesis of fetal vessels and in adhesion of fetal endothelial cells to the BM when LMα5 is absent [5]. These defects may be mechanistically related; the impaired adhesion suggests that endothelial cell migration should be inhibited due to reduced affinity for the BM, which endothelial cells likely use as a pathway for migration during angiogenesis. Endothelial cells originating from the allantois may bear a receptor with high affinity for LMα5, or LMα5 may bind and concentrate within the BM an adhesive or trophic factor required for promoting efficient angiogenesis. In any event, inefficient angiogenesis during the critical period of placentation leads to the observed defects and likely results in placental insufficiency. Further investigation into the mechanisms involved could lead to a better understanding of human placentation and of the regulation of angiogenesis in diverse tissues.

One important outcome of these studies with implications beyond the placenta and kidney is that the rescue of a deficiency in a BM need not always target all the cells that normally contribute to its synthesis. Given the accessibility of endothelial cells to potential nucleic acid, viral, or cell-based therapies via the bloodstream, they may be especially amenable to treatments that can influence the composition of vascular BMs throughout the body, whether in normal, diseased, or in cancerous tissue. And finally, our results represent another example of the cross-species compatibility of BM protein orthologs, suggesting that non-human BM proteins would likely be functional in the context of human tissues. This notion is also supported by a recent study of mice expressing human LMα5 from a BAC transgene [32].

## Materials and Methods

### Ethics Statement

All animal studies were approved by the Washington University Animal Studies Committee and performed in accordance with the NIH Guide for the Care and Use of Laboratory Animals.

### Genetically altered mice and administration of doxycycline

Mice carrying *LMα5* ^−^ and *LMα5^fl^* alleles and the tetO_7_-regulated hLMα5 cDNA have been previously described [5], [12], [27]. Purchased from The Jackson Laboratory were mice carrying the Sox2Cre transgene (stock #008454), expressed in epiblast when transmitted by sperm [25]; mice carrying the Tie2Cre transgene (stock #004128), expressed in endothelial cells [33]; and mice carrying the reverse tetracycline transactivator following a loxP-flanked neo/transcription termination signal inserted into the widely active *Rosa26* locus (stock #005572) [34]. When required to induce hLMα5 expression in endothelial cells, pregnant females were fed doxycycline-containing chow (0.15%; El Mel, Inc., St. Charles, MO) beginning on the day that a vaginal plug was observed (embryonic day 0.5) and continuing until the time of sacrifice.

### Antibodies

The antibodies or reagents that were used were as follows: rabbit anti-mouse LMα5 [35], mouse anti-hLMα5, clone 4C7 [30], rat anti-PECAM/CD31 (MEC 13.3; BD Pharmingen), rat anti-cytokeratin 8 (TROMA-1, Developmental Studies Hybridoma Bank, Iowa City, IA), rat anti-nidogen (clone ELM1; Millipore), rabbit anti-mouse laminin α1β1γ1 (LM-111) and Hoechst 33342 (Sigma, St. Louis, MO), rabbit anti-Wilms Tumor-1 (H-290; Santa Cruz Biotechnology, Santa Cruz, CA), and FITC- and Cy3-conjugated secondary antibodies (Molecular Probes, Eugene, OR).

### Immunofluorescence and electron microscopy

Frozen sections (8 µm) were fixed in 4% paraformaldehyde in PBS for 10 min and washed three times with PBS. After blocking with 5% normal goat serum in 1% BSA-PBS for 1 hr, the sections were incubated with the primary antibody overnight at 4°C, washed three times for 10 min with PBS, and incubated with secondary antibody for 30 min. Images were viewed with a Nikon Eclipse E800 microscope (Nikon Instruments Corp., Melville, NY USA) and captured with an Olympus DP2 digital camera using Olympus DP2-BSW software. Transmission electron microscopy was performed as described [36].

### Isolation of placental endothelial cells

Placentas from normal embryos were cleaned in Hank's balanced salt solution (HBSS, Sigma), cut into 1–3 mm pieces, and placed in 1 mg/ml collagenase at 37°C for 60 min with gentle pipetting every 15 min. Cell suspensions were filtered through a 70 µm cell strainer (BD Bioscience) and washed. After lysing red blood cells, isolated cells were incubated with phycoerythrin-conjugated PECAM/CD31 antibody (BD Pharmingen) for 30 min on ice. Prior to sorting, cells were washed, filtered through a 70 µm cell strainer, and resuspended with 1 mM EDTA/0.5%BSA/PBS. CD31-positive cells from placenta were sorted using a BD Aria II High Speed Cell Sorter, gated for high-level CD31 expression.

### RNA extraction and quantitative real-time PCR

Total RNA was isolated using RNeasy Mini or Micro Kit (Qiagen, Chatsworth, CA USA). Reverse transcription with oligo (dT) priming was performed using Superscript III (Invitrogen, Carlsbad, CA USA). The relative expression of each transcript was determined by quantitative real-time PCR in the fast mode (annealing and extending at 60°C) with a 7900 HT Fast Real-Time PCR System (Applied Biosystems, Forrest City, CA USA). Each well of the 96-well reaction plate contained a total volume of 20 µL with Fast Power SYBR Green PCR Master Mix (Applied Biosystems). The abundance of mRNA transcript was measured and normalized to glyceraldehyde 3-phosphate dehydrogenase (Gapdh). The primer sequences were: for LMα1, forward: 5′-CCAGTGACAAGGAGACAAAGC-3′, reverse: 5′-CACTCCGTAGGAATTTCTCAGC-3′; for LMα5, forward; 5′-TTGGTGCGTGTGGAGCGGGC-3′, reverse: 5′-ACTAGGAAGTGCCAGGGGCAG-3′; for LMβ1, forward: 5′-CGTGACCATCCAACTGGACCTGG-3′, reverse: 5′-CACGCCCCAAGCCTTCCCAA-3′; for LMβ2, forward: 5′-GACCTGTGCCATTGTGACCC-3′, reverse: 5′-GAGCTCTTGGCACTCAGAAC-3′; and for Gapdh, forward: 5′-AGGTCGGTGTGAACGGATTTG-3′, reverse: 5′-TGTAGACCATGTAGTTGAGGTCA-3′.

### Statistical analysis

Two-tailed, unpaired Student's *t*-tests were used to determine statistical difference. Differences were considered significant when the *P* value was <0.05.

## References

[1] Patton BL, Miner JH, Chiu AY, Sanes JR (1997). Distribution and function of laminins in the neuromuscular system of developing, adult, and mutant mice.. J Cell Biol.

[2] Noakes PG, Gautam M, Mudd J, Sanes JR, Merlie JP (1995). Aberrant differentiation of neuromuscular junctions in mice lacking s-laminin/laminin beta2.. Nature.

[3] Miner JH, Yurchenco PD (2004). Laminin functions in tissue morphogenesis.. Annu Rev Cell Dev Biol.

[4] Miner JH, Lewis RM, Sanes JR (1995). Molecular cloning of a novel laminin chain, α5, and widespread expression in adult mouse tissues.. J Biol Chem.

[5] Miner JH, Cunningham J, Sanes JR (1998). Roles for laminin in embryogenesis: Exencephaly, syndactyly, and placentopathy in mice lacking the laminin α5 chain.. J Cell Biol.

[6] Miner JH, Li C (2000). Defective glomerulogenesis in the absence of laminin α5 demonstrates a developmental role for the kidney glomerular basement membrane.. Dev Biol.

[7] Nguyen NM, Miner JH, Pierce RA, Senior RM (2002). Laminin alpha 5 is required for lobar septation and visceral pleural basement membrane formation in the developing mouse lung.. Dev Biol.

[8] Fukumoto S, Miner JH, Ida H, Fukumoto E, Yuasa K (2006). Laminin alpha5 is required for dental epithelium growth and polarity and the development of tooth bud and shape.. J Biol Chem.

[9] Coles EG, Gammill LS, Miner JH, Bronner-Fraser M (2006). Abnormalities in neural crest cell migration in laminin alpha5 mutant mice.. Dev Biol.

[10] Gao J, Derouen MC, Chen CH, Nguyen M, Nguyen NT (2008). Laminin-511 is an epithelial message promoting dermal papilla development and function during early hair morphogenesis.. Genes Dev.

[11] Mahoney ZX, Stappenbeck TS, Miner JH (2008). Laminin {alpha}5 influences the architecture of the mouse small intestine mucosa.. J Cell Sci.

[12] Nguyen NM, Kelley DG, Schlueter JA, Meyer MJ, Senior RM (2005). Epithelial laminin alpha5 is necessary for distal epithelial cell maturation, VEGF production, and alveolization in the developing murine lung.. Dev Biol.

[13] Rebustini IT, Patel VN, Stewart JS, Layvey A, Georges-Labouesse E (2007). Laminin alpha5 is necessary for submandibular gland epithelial morphogenesis and influences FGFR expression through beta1 integrin signaling.. Dev Biol.

[14] St. John PL, Abrahamson DR (2001). Glomerular endothelial cells and podocytes jointly synthesize laminin-1 and -11 chains.. Kidney Int.

[15] Watson ED, Cross JC (2005). Development of structures and transport functions in the mouse placenta.. Physiology (Bethesda).

[16] Simmons DG, Natale DR, Begay V, Hughes M, Leutz A (2008). Early patterning of the chorion leads to the trilaminar trophoblast cell structure in the placental labyrinth.. Development.

[17] Tiger C-F, Champliaud M-F, Pedrosa-Domellof F, Thornell L-E, Ekblom P (1997). Presence of laminin α5 chain and lack of laminin α1 chain during human muscle development and in muscular dystrophies.. J Biol Chem.

[18] Abrahamson DR, St John PL, Isom K, Robert B, Miner JH (2007). Partial rescue of glomerular laminin alpha5 mutations by wild-type endothelia produce hybrid glomeruli.. J Am Soc Nephrol.

[19] Hallmann R, Horn N, Selg M, Wendler O, Pausch F (2005). Expression and function of laminins in the embryonic and mature vasculature.. Physiol Rev.

[20] Kikkawa Y, Miner JH (2006). Molecular dissection of laminin alpha 5 in vivo reveals separable domain-specific roles in embryonic development and kidney function.. Dev Biol.

[21] Miner JH, Go G, Cunningham J, Patton BL, Jarad G (2006). Transgenic isolation of skeletal muscle and kidney defects in laminin beta2 mutant mice: implications for Pierson syndrome.. Development.

[22] Miyagoe Y, Hanaoka K, Nonaka I, Hayasaka M, Nabeshima Y (1997). Laminin alpha2 chain-null mutant mice by targeted disruption of the Lama2 gene: a new model of merosin (laminin 2)-deficient congenital muscular dystrophy.. FEBS Lett.

[23] Thyboll J, Kortesmaa J, Cao R, Soininen R, Wang L (2002). Deletion of the laminin alpha4 chain leads to impaired microvessel maturation.. Mol Cell Biol.

[24] Wang J, Hoshijima M, Lam J, Zhou Z, Jokiel A (2006). Cardiomyopathy associated with microcirculation dysfunction in laminin alpha4 chain-deficient mice.. J Biol Chem.

[25] Hayashi S, Lewis P, Pevny L, McMahon AP (2002). Efficient gene modulation in mouse epiblast using a Sox2Cre transgenic mouse strain.. Mech Dev.

[26] Shaut CA, Keene DR, Sorensen LK, Li DY, Stadler HS (2008). HOXA13 Is essential for placental vascular patterning and labyrinth endothelial specification.. PLoS Genet.

[27] Goldberg S, Adair-Kirk TL, Senior RM, Miner JH (2010). Maintenance of glomerular filtration barrier integrity requires laminin alpha5.. J Am Soc Nephrol.

[28] Wulff C, Weigand M, Kreienberg R, Fraser HM (2003). Angiogenesis during primate placentation in health and disease.. Reproduction.

[29] Carter AM (2007). Animal models of human placentation–a review.. Placenta 28 Suppl A.

[30] Engvall E, Davis GE, Dickerson K, Ruoslahti E, Varon S (1986). Mapping of domains in human laminin using monoclonal antibodies: localization of the neurite-promoting site.. J Cell Biol.

[31] Korhonen M, Virtanen I (2001). Immunohistochemical localization of laminin and fibronectin isoforms in human placental villi.. J Histochem Cytochem.

[32] Steenhard BM, Zelenchuk A, Stroganova L, Isom K, St John PL (2011). Transgenic expression of human LAMA5 suppresses murine Lama5 mRNA and laminin alpha5 protein deposition.. PLoS One.

[33] Koni PA, Joshi SK, Temann UA, Olson D, Burkly L (2001). Conditional vascular cell adhesion molecule 1 deletion in mice: impaired lymphocyte migration to bone marrow.. J Exp Med.

[34] Belteki G, Haigh J, Kabacs N, Haigh K, Sison K (2005). Conditional and inducible transgene expression in mice through the combinatorial use of Cre-mediated recombination and tetracycline induction.. Nucleic Acids Res.

[35] Miner JH, Patton BL, Lentz SI, Gilbert DJ, Snider WD (1997). The laminin alpha chains: expression, developmental transitions, and chromosomal locations of alpha1–5, identification of heterotrimeric laminins 8–11, and cloning of a novel alpha3 isoform.. J Cell Biol.

[36] Jarad G, Cunningham J, Shaw AS, Miner JH (2006). Proteinuria precedes podocyte abnormalities in Lamb2−/− mice, implicating the glomerular basement membrane as an albumin barrier.. J Clin Invest.

